# Usage of artificial intelligence and virtual reality in medical studies

**DOI:** 10.12669/pjms.38.4.5910

**Published:** 2022

**Authors:** Ambreen Usmani, Manal Imran, Quratulain Javaid

**Affiliations:** 1 Prof Ambreen Usmani, MBBS, MPhil (Anatomy), MCPH (HPE), PGD (Bioethics), PhD (Anatomy). Professor of Anatomy, Deputy Director Medical Education, Bahria University Medical and Dental College, Karachi, Pakistan; 2 Ms. Manal Imran, 1^st^ Year MBBS Dow University of Health Sciences; 3 Quratulain Javaid, MBBS, PGD (Bioethics), MPhil (Anatomy). Assistant Professor of Anatomy, Bahria University Medical and Dental College, Karachi, Pakistan

Human beings of the modern age have become a part of the digitized life.^1^ The individuals of the contemporary world have adopted novel ways of acquiring knowledge and proficiency in skills by using Artificial Intelligence (AI) and Virtual Reality (VR) as their teaching and learning modalities. AI is the simulation of human intelligence processes by machines, specifically by computer systems. Various applications of this technology include expert systems, natural language processing, speech recognition and machine vision. On the other hand, VR is the computer generated simulation of a three-dimensional image or environment that an individual can interact with in a seemingly real or physical way. A person can do so by using special electronic equipment, such as a helmet with a screen mounted in it or gloves fitted with sensors.^2^,^3^

As the world is increasingly incorporating the use of technology in everyday lives, it is important that the use of AI and VR are assimilated in medical studies so the field can progress at the same pace as the rapidly developing world. A study conducted in Brussels has mentioned the diagnostic significance of artificial intelligence in detection of atrial fibrillation, glucose levels, glomerular filtration rate, epilepsy, etc.^4^ Several studies have reported that before major complicated surgical procedures surgeon mimic their surgeries using such technologies and then these procedures are performed in real time which reduces the risk of complications and failure.^5^

Due to advancing technology, medical education has been involved in planning new strategies with a lot of focus on simulation teaching and other similar modalities.^6^ These teaching strategies enable the student to go through real life scenarios and solve them. They also feel the same urgency while doing procedures-examples are spontaneous vaginal delivery and complications that can be faced in real time scenarios. This has also trickled down to teaching in the same way as we are preparing students for the future in which a major chunk will constitute the use of AI and VR. Literature shows that studies have supported the introduction of AI and VR in teaching basic science subjects like Anatomy, Physiology, Biochemistry, Pathology etc. as well as clinical subjects such as Medicine, Surgery, Obstetrics & Gynaecology, and Paediatrics etc.^7^ Complete shifting from simple multimedia and board teaching will not be possible at the present however AI and VR will augment the already running technology though some aspects may become obsolete completely.^8^

Alharbi et al in their research have documented the fruitful effects of virtual reality in teaching anatomy. The new modality offered better learning as compared to the conventional teaching methods.^9^ Teaching medical sciences with less class room teaching and more case based simulated learning is the future learning for the students e.g. the Anatomy dissection table shows the students to dissect human bodies layer by layer and this can be taught to several students at the same time where they can sit and observe in small groups on smart boards. The anatomy dissection table serves as a source of innovative learning by the help of digital softwares. They can also practice themselves several times whereas in cadavers they could only dissect once and then study on the prosected parts. Although there is no substitute for real time dissection but the newer tool can aid in understanding the subject by the help of digital visualization.^10^ Also, with scarcity of cadavers it is not possible for all students to perform full dissection, also if the future holds promise that treatment will soon be dependent on AI then it is beneficial for the students to be handy with such technology and softwares.^7^

Different case scenarios can be added in the softwares enhancing the interest of the students. The students themselves can be proactive and learn medical sciences virtually. Similarly, virtual laboratories can be created to depict real life lab experiments and the outcome of the experiment or result of the procedure can be seen by the student which can also be recorded to point out mistakes so as to self-correct themselves. The students will become more responsible for their learning and not be dependent for help all the time.^3^,^6^,^7^ The capability to pile hefty data repositories on the computer is problematic causing in handling of accommodation of knowledge data on the “cloud AI and machine learning platform services known as AI Platforms as a Service (PaaS) are used”. In health, recent AI expansions that are displaying hopeful results are data driven approaches, specially for hospital based doctors and clinicians who encounter applications such as interpretation and analysis of image in radiology. Deep Learning driven by computer aided diagnosis is a type of representation learning method that can discover representations of data automatically by transforming the input information into multiple layers of abstractions in a deep neural network architecture. By training with a large data set and an appropriate cost function, the multiple layers of weights in the deep neural network are iteratively updated, resulting in a complex mathematical model that can extract relevant features from the input data with high selectivity and invariance. Deep Learning has taken the world to higher expansions in several self-generated or assisted by computer-in built knowledge including “target detection and characterization, speech and text recognition, face recognition, autonomous vehicles, smart devices, and robotics”.^7^

The curriculum of medical education should cater to the need of the advancing digitalized world.^11^ A study by Masters et al documented that the growing phenomenon of AI and VR should be taught both to the teachers and the students keeping in mind their implications on the medical education.^2^ Social media stages and encounters, including health communities online have become widespread sources for people of all intellect to join and exchange support. Hallak J et al emphasized AI applications in ophthalmology during COVID-19 pandemic. They used “natural language processing and data integration methods, topic modelling” on more than 200 cases. The authors summarized that COVID-19 is already significantly affecting the way we are providing health and patient care. This state has played a role in bringing the people and health care providers closer to AI applications and telemedicine. The authors emphasized that AI applications should be used for research and medical education at undergraduate level as it is already being used for patient care for protecting the healthcare environment.^8^

With the apace scientific revolution, one has to plan according to the needs of the contemporary world.^12^ There is a dire need to focus on the novel subject of artificial intelligence other than the routine subjects of clinical and biomedical sciences. This will enable the students of this era to face the challenges and advancements of the transformed health care environment.^13^ The medical universities should prepare the students from year one of their studies. AI and VR promise to the world a higher level of treatment with less infection transfer and must be incorporated in the curriculum of all medical colleges. The future of our generations lies in the hands of the present day academicians so the earlier they are drenched in the technology pool, the better it is for them to get used to the innovations around them.^14^,^15^
